# Evidence of COMT dysfunction in the olfactory bulb in Parkinson’s disease

**DOI:** 10.1007/s00401-025-02861-y

**Published:** 2025-03-01

**Authors:** Leah C. Beauchamp, Laura J. Ellett, Sydney M. A. Juan, Xiang M. Liu, Cameron P. J. Hunt, Clare L. Parish, Laura H. Jacobson, Claire E. Shepherd, Glenda M. Halliday, Ashley I. Bush, Laura J. Vella, David I. Finkelstein, Kevin J. Barnham

**Affiliations:** 1 Ann Romney Center for Neurologic Diseases, Brigham and Women’s Hospital, Harvard Medical School, Boston, MA 02115 USA; 2 The Florey Institute of Neuroscience and Mental Health, and The University of Melbourne, Parkville, VIC 3052 Australia; 3 Department of Biochemistry and Pharmacology, University of Melbourne, Parkville, VIC 3052 Australia; 4 Neuroscience Research Australia, Sydney, NSW 2031 Australia; 5 Faculty of Medicine and Health, School of Medical Sciences, University of Sydney Brain and Mind Centre, Camperdown, NSW Australia; 6 Department of Surgery, University of Melbourne, Parkville, VIC 3010 Australia

**Keywords:** Parkinson’s disease, Hyposmia, Dopamine, Catechol-*O*-methyltransferase, Olfaction

## Abstract

Hyposmia is one of the most prevalent non-motor symptoms of Parkinson’s disease and antecedes motor dysfunction by up to a decade. However, the underlying pathophysiology remains poorly understood. In this study, we investigated the mechanisms of dopamine metabolism in post-mortem olfactory bulbs from ten Parkinson’s disease and ten neurologic control subjects. In contrast to the loss of dopaminergic neurons in the midbrain, we observed an increase in tyrosine hydroxylase-positive neurons in the Parkinson’s disease olfactory bulb, suggesting a potential role for dopamine in the hyposmia associated with the condition. Using immunohistochemistry, high-performance liquid chromatography, western blot, and enzyme-linked immunosorbent assays, we demonstrate a reduction in catechol-*O*-methyltransferase catabolism of dopamine to homovanillic acid, potentially due to a depletion of the methyl donor substrate *S*-adenosyl methionine. We hypothesized that reduction in catechol-*O*-methyltransferase activity would result in increased dopamine occupation of the D_2_ receptor, and consequent inhibition of olfactory processing. Next, we conducted pharmacological interventions to modify dopamine dynamics in hyposmic tau knockout mice, which exhibit altered dopamine metabolism. Our hypothesis was supported by the observation that the D_2_ receptor antagonist haloperidol temporarily alleviated olfactory deficits in these tau knockout mice. This study implicates a potential role of catechol-*O*-methyltransferase-mediated dopamine metabolism in the early olfactory impairments associated with Parkinson’s disease.

**Supplementary Information:**

The online version contains supplementary material available at 10.1007/s00401-025-02861-y.

## Introduction

Parkinson’s disease (PD) is a progressive neurodegenerative disorder that is primarily associated with motor impairments. Diagnosis of PD is reliant on clinical assessment of movement symptoms which manifest as many as 10–20 years into the neurodegenerative process [3, 5, 56]. The reliance on motor impairment for diagnosis and the heterogenous presentation of symptoms results in a high mis-diagnosis rate in PD, particularly during the first 5 years of motor manifestations [1]. In the pre-motor (prodromal) phase of the disease, various non-motor symptoms can emerge, such as hyposmia, gastrointestinal dysfunction, sleep disturbances, and depression [37]. The early occurrence of these symptoms presents an opportunity for earlier identification of patients, and they are increasingly being studied as potential biomarkers for underlying neurodegeneration that has yet to manifest as motor symptoms [53].

Hyposmia occurs in up to 90% of idiopathic PD cases and can precede motor dysfunction by as much as a decade; however, the underlying pathophysiology of this prominent symptom remains unclear [16]. Early neuropathological changes in the olfactory bulb and anterior olfactory nucleus (AON) are observed in PD, followed by alterations in the olfactory cortex and limbic structures as the disease progresses. This suggests that PD pathology may initially involve the primary olfactory structures [7].

Olfactory pathologies in PD that have been reported to date include non-dopaminergic neurodegeneration and α-synuclein pathology in the primary olfactory structures, with these changes correlating to the duration of the disease. Additionally, tau inclusions in the AON have been linked to α-synuclein Braak staging [12, 47, 64]. Despite these findings indicating a spread of neuropathology, there is no clear correlation between hyposmia and either disease duration or clinical severity. This implies that olfactory deficits may be independent of the progressive neuropathology and motor impairments associated with PD [17, 27, 50, 60].

Unlike motor symptoms, dopamine replacement pharmacotherapies do not improve olfactory test scores [54]. This observation aligns with findings that glutamatergic bulbar projection neurons are primarily affected by Lewy Body pathology, while the local dopaminergic periglomerular neurons remain largely unaffected [20, 49, 55, 65]. Dopamine synthesis begins with the amino acid tyrosine, which is converted into l-DOPA by the enzyme tyrosine hydroxylase. l-DOPA is then decarboxylated to form dopamine. Metabolism of dopamine involves enzymatic breakdown by monoamine oxidase (MAO) and catechol-*O*-methyltransferase (COMT), producing metabolites including 3,4-Dihydroxyphenylacetic acid (DOPAC) and homovanillic acid (HVA). Interestingly, there is a notable increase in the expression of tyrosine hydroxylase (TH), the rate-limiting enzyme in dopamine synthesis, within the periglomerular cells of the olfactory bulb, accompanied by a rise in the number of dopaminergic periglomerular cells [30, 45]. In light of these findings, it has been suggested that elevated dopamine levels may contribute to olfactory dysfunction in PD [30]. Increased dopaminergic activity in the olfactory bulb could inhibit the transmission of olfactory signals, as dopamine acts as an inhibitor of communication between olfactory receptor cells and mitral cells within the olfactory glomeruli, thereby promoting hyposmia [19, 36, 58].

These findings suggest a role for dopamine in PD-related hyposmia; however, the specifics of dopaminergic changes in the olfactory system have yet to be explored. We aimed to investigate dopamine synthesis and metabolism in post-mortem olfactory bulbs from individuals with PD and neurological controls. Following this, we pharmacologically altered the dopaminergic system in tau ablated mice, a model of parkinsonism that exhibits olfactory deficits before motor symptoms appear [4]. This approach allowed us to characterize the effect of dopamine metabolism on olfaction. Understanding the biological basis of hyposmia in PD has the potential to inform future diagnostic strategies.

## Methods

This study involved the analysis of both frozen and formalin-fixed human olfactory bulbs, followed by in vivo experiments in tau knockout mice and further examination of fresh-frozen murine olfactory tissue. In both human and murine olfactory bulbs, dopaminergic pathways were assessed using multiple methods: (1) Western blot to measure tyrosine hydroxylase levels (a marker of dopamine synthesis), (2) immunohistochemistry to quantify dopaminergic neurons, (3) high-performance liquid chromatography to determine levels of dopamine and its metabolites (DOPAC and HVA), (4) ELISA to measure the levels of COMT and MAO activity (key enzymes involved in dopamine metabolism), and (5) ICP-MS and ELISA to measure magnesium and *S*-adenosylmethionine (required for COMT activation), respectively.

### Human and murine tissue analysis

#### Post-mortem tissue

Both olfactory bulbs were collected from ten neuropathologically confirmed Parkinson’s disease and ten neurological controls (Table 1). Neurological controls are defined as individuals without a neurological diagnosis and a Braak Lewy score of 0. For PD cases, inclusion was based on *post-mortem* confirmation of Braak Lewy pathology supporting the clinical PD diagnosis. Fixed and frozen tissue was acquired through the New South Wales Brain Bank under human ethics approval from University of Melbourne (HREC: 1750801). New South Wales Brain Bank pathologically confirmed PD diagnosis, with all PD tissue reported as Braak Lewy stage 5 or 6 [8]. Common co-pathologies were also analyzed using the ABC score for amyloid plaque, neurofibrillary tangle, and neuritic plaques according to standardized criteria [31], and assessed for ‘AD likelihood’ based on the ABC score. This data was not available for two male and two female control subjects, noted by NA in Table 1. One bulb was snap frozen and stored at − 80 °C, and the contralateral bulb was formalin fixed.

**Table 1:** Donor and tissue characteristics

	Age	Sex	DD (years)	Braak Lewy stage	A score (amyloid plaque)	B score (NFT)	C score (neuritic plaques)	AD likelihood	Dopaminergic medications
PD	84	M	12	5	0	0	0	Not AD	l-Dopa, carbidopa, entacapone
	82	M	22	6	1	0	0	Low AD	l-Dopa, benserazide
	81	F	22	6	0	1	0	Not AD	l-Dopa, carbidopa
	71	M	15	5	0	1	0	Not AD	l-Dopa, carbidopa, entacapone, pramipexole
	82	F	8	5	0	2	0	Not AD	l-Dopa, benserazide
	92	M	8	6	3	1	3	Low AD	N/A
	93	F	32	6	1	2	0	Not AD	l-Dopa, carbidopa
	78	M	18	6	1	0	0	Low AD	l-Dopa, carbidopa, entacapone, pramipexole
	80	F	7	5	0	1	0	Not AD	l-Dopa, carbidopa, pramipexole
	88	F	16	5	2	1	0	Low AD	Anticholinergic and entacapone
NC	79	M		0	0	1	0	Not AD	
	89	F		0	1	1	0	Not AD	
	88	F		0	2	1	3	Low AD	
	84	M		0	2	1	1	Low AD	
	84	M		0	1	0	0	Low AD	
	80	F		0	1	1	0	Low AD	
	73	M		NA	NA	NA	NA	NA	
	88	M		NA	NA	NA	NA	NA	
	85	F		NA	NA	NA	NA	NA	
	86	F		NA	NA	NA	NA	NA	

*AD* Alzheimer’s disease, *DD* disease duration, *F* female, *M* male, *NA *not available, *NC* neurological control, *NFT* neurofibrillary tangle, *PD* Parkinson’s disease

#### SDS-PAGE and immunoblot analysis

10% w/v homogenates were prepared in radioimmunoprecipitation assay (RIPA) lysis buffer (150 mM NaCl, 1% NP-40, 0.5% deoxycholic acid, 50 mM Tris) with protease and phosphatase inhibitors (Complete Mini Protease Inhibitor Cocktail and PhosSTOP Phosphatase Inhibitor Cocktail, Roche Diagnostic; Australia) and butylated hydroxytoluene (BHT) (buffer volume (µL) determined by 9 × sample weight (mg)) using a probe sonicator (Digital Sonifier, Branson; USA) for 20 s. Homogenate was clarified at 10,000 g at 4 °C for 20 min and the clarified supernatant collected. The total protein concentrations were determined using the bicinchoninic acid (BCA) assay (Pierce; USA) according to the manufacturer’s directions and protein concentrations were normalized in RIPA lysis buffer prior to standard gel electrophoresis.

Proteins were visualized using either fluorescence with the Licor Odyssey fc system (Licor; Australia) and analyzed via signal intensities (ImageStudio 5.2, Licor; Australia) or enhanced chemiluminescence. Human samples were normalized to β-actin. Animal samples were normalized to total protein analysis performed via the ChemiDoc system (BioRad; USA). Antibodies and antibody dilutions provided in Supplementary Table 1.

#### Histology

Human: 5-micron thick sections were prepared from paraffin embedded fixed tissues and processed for immunohistochemical (IHC) analysis. Sections were de-paraffinized and non-specific binding sites were blocked with normal goat serum.

Mouse: 20-micron thick frozen sections were sectioned on a cryostat and non-specific binding sites were blocked with normal goat serum.

Sections were then incubated with an anti-TH antibody overnight followed by a PBS rinse and incubation with biotin conjugated secondary antibody for 3 h at room temperature. Sections were then incubated in avidin peroxidase solution (A-7419, Sigma; USA) for 1 h. Finally, sections were incubated in diaminobenzidine tetrahydrochloride (D5637, Sigma; USA) with 3% H_2_O_2_. Sections were rinsed, counter stained with Neutral Red, dehydrated, and cover slipped. The quantitation represents the total number of positive cells counted divided by the area [the area is: total area of the region traced/area of the counting frame, where the counting frame is 30 μm (X-axis) by 30 μm (Y axis)]. TH^+^ neurons were quantified within the glomerular layer of the olfactory bulbs, the only layer of the olfactory bulb where dopaminergic neurons are present. For human samples, a 1:10 series were collected for quantification, with a total of 8 sections per sample. For murine samples, a 1:5 series were collected for quantification, with a total of 6 sections per sample. Antibodies and antibody dilutions are provided in Supplementary Table 1.

#### Inductively coupled plasma mass spectrometry

Inductively coupled plasma mass-spectrometry (ICP-MS) was performed as previously reported [38]. Briefly, samples were lyophilized and nitric acid (65% Suprapur, Merck; USA) was added for a 6-h digestion at RT. Samples were heated at 90 °C for 20 min followed by the addition of hydrogen peroxide (30% Aristart, BDH; UAE). Samples were left at room temperature for 30 min before heating again for a further 15 min at 70 °C. The average reduced volume was determined, and the samples were further diluted with 1% HNO_3_ diluent. Measurements were made using an Agilent 7700 series ICP-MS instrument under routine multi-element operating conditions using a Helium Reaction Gas Cell. The instrument was calibrated using 0, 5, 10, 50, 100, and 500 ppb of certified multi-element ICP-MS standard calibration solutions (ICP-MS-CAL2-1, ICP-MS-CAL-3, and ICP-MS-CAL-4, Accustandard; USA) for a range of elements. Certified internal standard solution containing 20 ppb of Yttrium (Y89) as an internal control (ICP-MS-IS-MIX1-1, Accustandard; USA) was used.

#### High performance liquid chromatography with electrochemical detection

Dopamine, 3,4-dihydroxyphenylacetic acid (DOPAC) and homovanillic acid (HVA) levels were measured using reverse phase liquid chromatography with electrochemical detection as previously described [44]. Human and mouse olfactory bulbs were dissected, weighed, and placed in 200 µL 0.4 M perchloric acid (HCIO_4_) containing 0.05% sodium metabisulphate (Na_2_S_2_O_5_) and 0.01% disodium EDTA. The tissue was homogenized, centrifuged at 10,500 g for 10 min and filtered through MiniSpin filters (POCD Scientific; Australia) for 3 min before being injected into the HPLC. For each sample, 5 µL was injected by a cooled autosampler (SIL 20A, Shimadzu, Australia) and Shimadzu LC-AT pump on to a reverse-phase C18 column (4.6 mm diameter, 150 mm length; CHROMPACK, UK) coupled with an electrochemical detector (Decade II, Antec Leyden, Australia). The mobile phase, (KH2PO4, 70 mM; EDTA di-sodium salt, 0.5 mM; octane-sulphonic acid, sodium salt, 8 mM; with 17% HPLC grade methanol, pH 3) was delivered at a flow rate of 500 mL min^−1^).

#### Enzyme-linked immunosorbent assays

For all ELISAs, total protein concentrations were determined using the BCA assay (Pierce; USA) according to the manufacturer’s directions and protein concentrations were normalized in provided diluent buffer. Catechol-*O*-methyltransferase (COMT) levels (ab213766, Abcam; USA); *S*-adenosylmethionine (SAMe) levels (Cell Biolabs Inc.; USA); human D_2_ receptor (D_2_R) (A74190, antibodies.com; USA); monoamine oxidase (MAO) activity was analyzed using a commercially available ELISA kit (ab241031), Abcam; USA) according to manufacturer’s instructions.

### In vivo experimentation

Animals used in this study are the tau knockout (tau KO) mice on an Sv129/B6 background, originally described by Dawson et al. [14]. All mice were genotyped using a standardized polymerase chain reaction assay for tail DNA (Transnetyx Inc., USA). Mice were group-housed in standard transparent individually ventilated cages (29.5 × 16 × 13 cm) on sawdust under a 12 h light/dark cycle (lights on at 0700 h). Rodent chow and water were available ad libitum. Experimentation was performed in accordance with the Prevention of Cruelty to Animals Act (2004), under the guidance of the National Health and Medical Research Council Code of Practice for the Care and Use of Animals for Experimental Purposes in Australia (2013). Individual experiments were approved by The Florey Animal Ethics Committee (AEC number: 19-052).

#### Odor detection test

The odor detection test (ODT) was adapted from a previously described protocol [48]. Mice were habituated to vehicle canisters for 3 days prior to testing. The test day (day 4) comprised of three 5-min trials (1 h inter-trial interval) performed in the home cage in which the mice were exposed to two visually identical canisters; one vehicle (400 µL, MilliQ water + 0.1% Tween20) and one novel odor canister of either 0 (vehicle) or 1:10^4^ dilutions [400 µL, MilliQ water + 0.1% Tween20 + orange, peppermint, or juniper berry essential oil (In Essence; Australia)]. Animals were filmed and videos were manually scored (the scorer was blinded to experimental conditions), and the percentage of investigation time was calculated based on: (*time spent investigating novel odor canister / time spent investigating both canisters*) × *100.* Normal mice will spend more time investigating a novel odor, as such this test determines the concentration at which mice can detect a novel odor by comparing the time spent investigating the two canisters.

#### Preparation of pharmacological agents

Cocaine hydrochloride (Johnson Matthey Macfarlan Smith, UK) was diluted in saline 30 min before injection. Haloperidol (Sigma Aldrich, Aus) was diluted in 0.1 M HCl and neutralized to pH 7.2 1 h before injection. *S*-adenosylmethionine (Sigma Aldrich, Australia) was stored at − 80 °C and aliquots were diluted in double-distilled water (ddH_2_O) 30 min before oral gavage dosing.

#### Experimental timelines

Cocaine and haloperidol experiment. Day 1: 8-month-old WT and tau KO animals underwent a baseline Odor Detection Test (ODT) (peppermint scent). Day 8: animals were randomly assigned a dosing group and received a single intraperitoneal (i.p.) injection of either cocaine (20 mg/kg), haloperidol (0.33 mg/kg) or control (saline). Post-injection, animals were isolated for 1 h in a clean cage before undergoing a second ODT (juniper berry scent). Day 9: After a 24 h flush out time, animals underwent a final ODT (orange scent). Scents were changed throughout the experiment to avoid olfactory memory confounds.

#### Transcardiac perfusion and tissue collection

At the conclusion of behavioral tests, animals were terminally anaesthetized using 100 mg/kg intraperitoneal pentobarbitone (Virbac, Australia) injection and transcardiac perfused with Dulbecco’s phosphate buffered saline (D-PBS) (Gibco, Australia) containing 55.6 mg/L heparin (Sigma Aldrich, Aus) with approximately 3 times their blood volume (7% of body weight). Olfactory bulbs were extracted and frozen on dry ice before being stored at − 80 °C for later processing.

### Statistical analysis

For all statistical analyses and graphing, the software package GraphPad Prism (version 6.05 for Windows) was used. ANOVA *P* values reported are from post hoc comparisons generated only when ANOVA terms were significant. One-sample t-tests were also performed for ODT results to determine if the animals could detect odors at specified concentrations (independent of genotype), as evidenced by spending significantly more than 50% of the investigation time with a novel odorous canister. An investigation time of 50% represents chance, indicating an animal cannot differentiate between the canisters, i.e. cannot detect an odor. For all analyses, *P* < 0.05 was considered statistically significant.

## Results

### Parkinson’s disease pathology is observed in human olfactory bulbs

Donor and post-mortem tissue characteristics are reported in Table 1. There was an equal distribution of sex in both PD and NC groups (5 males and 5 females per group). The age of the PD donors was lower than the NC, although this was not significant (79.1 ± 5.6 and 83.6 ± 5.0 respectively, *P* = 0.07). The average disease duration was 16.0 ± 7.9 years, and there was no difference in average post-mortem delay (PMD) between the PD and NC groups (21.0 ± 12.7 and 21.0 ± 11.9 h respectively, *P* = 0.99) (Supplementary Table 2). All PD cases were pathologically confirmed, with Braak Lewy stage 5 and 6 in each PD case, and no HC cases presenting with Lewy pathology. ABC scores revealed common Alzheimer’s co-pathology in both PD and HC samples, with 4/10 PD cases and 4/6 HC cases characterized as ‘low AD likelihood’. 8/10 PD cases were on dopaminergic therapy (one case had no medication history available, and one case was on an anti-cholinergic therapy), including l-dopa and pramipexole. All donors on dopamine therapy were co-treated with peripheral DOPA decarboxylase inhibitors (carbidopa or benserazide), and four PD donors were on entacapone, a peripherally acting COMT inhibitor. PD-related neuropathology was observed in PD olfactory bulbs including increased α-synuclein (*P* = 0.03) and DJ1 (*P* = 0.0004) protein levels, elevated iron (a key regulator of tyrosine hydroxylase function [26]) (*P* = 0.07) as determined by ICP-MS, as well as the presence of α-synuclein inclusions (Supplementary Fig. 1).

### Dopamine metabolism is altered in Parkinson’s disease olfactory bulbs

**Fig. 1:**
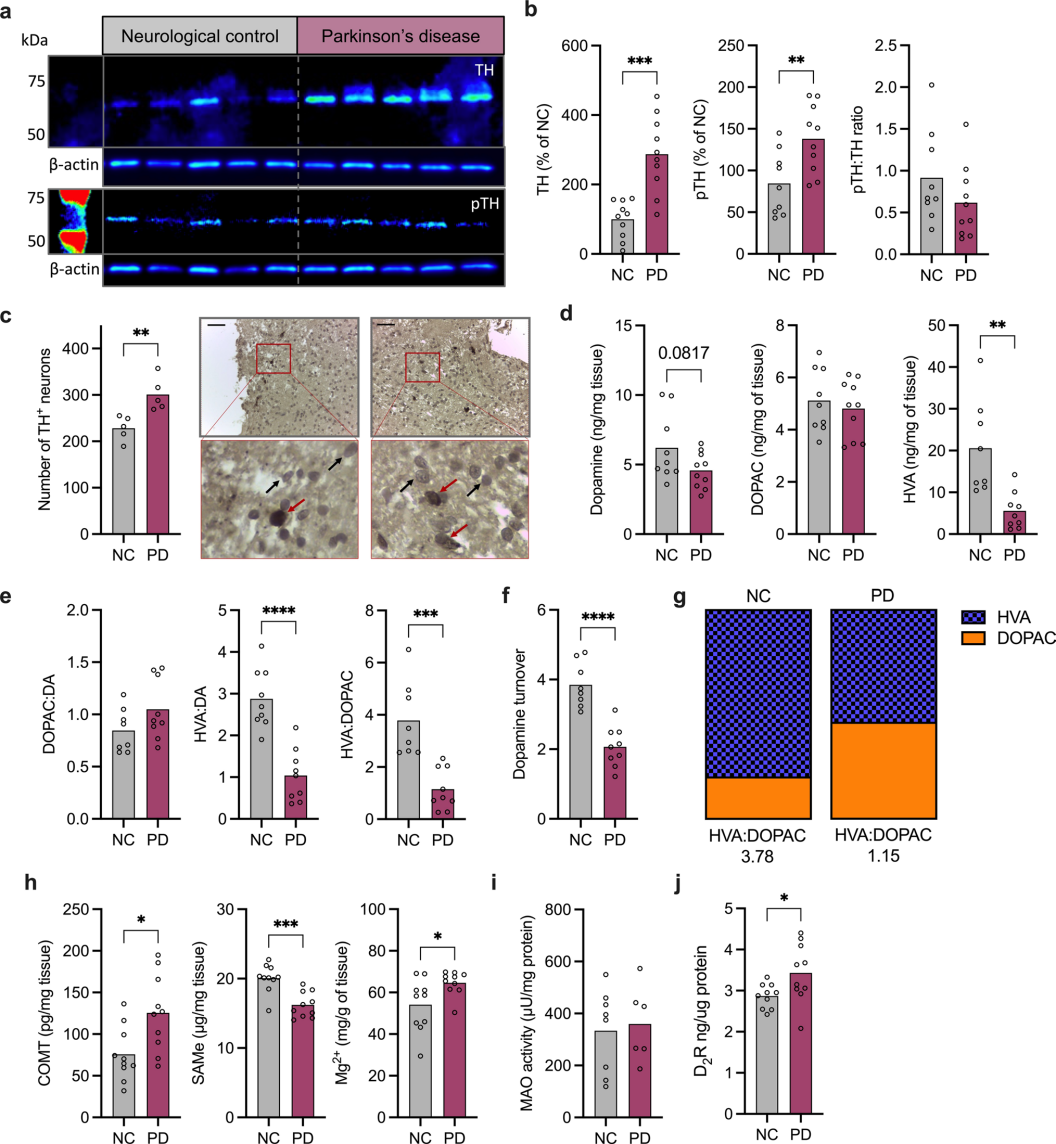
Dopamine metabolism is altered in the PD olfactory bulb. **a** Representative immunoblots of olfactory bulb lysates stained for tyrosine hydroxylase (TH), pTH (ser40) and β-actin. **b** Quantification of TH and pTH immunoblot densitometry presented as % of neurological control, and ratio of pTH:TH. **c** Quantification of TH^+^ neurons, representative images of TH-stained olfactory bulb sections from one NC and one PD sample; red arrows indicate TH^+^ neurons, scale bar represents 200 μm. **d** Dopamine, 3,4-dihydroxyphenylacetic acid (DOPAC), and homovanillic acid (HVA) concentration determined by HPLC-ED. **e** Ratio of DOPAC:dopamine, HVA:DOPAC and HVA:dopamine in olfactory bulb tissue. **f** Dopamine turnover (DOPAC + HVA/dopamine). **g** Proportion of HVA to DOPAC. **h** Catechol-*O*-methyltransferase (COMT) protein level, *S*-adenosylmethionine (SAMe) protein levels, and magnesium levels in tissue lysate. **i** Monoamine oxidase activity level. **j** D_2_ receptor protein level. NC (*N* = 10) and PD (*N* = 10). Analyses performed by Students’ T Test; *P < 0.05, ** *P* < 0.01, ***P < 0.001, ****P < 0.0001

Tyrosine hydroxylase (TH) is the rate-limiting enzyme in dopamine synthesis, responsible for converting l-tyrosine to l-DOPA, which is then available for conversion to dopamine [13]. TH and the active phosphorylated form (pTH; pSer40) were measured by immunoblot. TH and pTH levels were both increased in the PD olfactory bulb as compared to NC (*P* = 0.0001 and *P* = 0.009, respectively) and the ratio of pSer40TH:TH reduced, though not significantly (Fig. 1a and b). Consistent with the immunoblot data, there was a significant increase in the number of TH positive (TH^+^) neurons in the PD olfactory bulb (*P* = 0.007), as determined by immunohistochemistry (Fig. 1c).

Next, dopamine synthesis and metabolism were investigated using HPLC-ED. Intracellularly, dopamine is catabolized to 3,4-dihydroxyphenylacetic acid (DOPAC) by monoamine oxidase, before conversion to homovanillic acid (HVA). Extracellularly, dopamine is converted to 3-methoxytyramine by COMT, before conversion to HVA [33]. There was no difference in the concentration of dopamine or DOPAC between NC and PD, however there were significantly reduced levels of HVA (*P* = 0.002) (Fig. 1d). This significant reduction in HVA resulted in a decreased ratio of HVA:DOPAC (*P* = 0.0003) and HVA:dopamine (*P* < 0.0001) (Fig. 1e), and there was a significant reduction in dopamine turnover (DOPAC + HVA/dopamine) in PD olfactory bulbs (*P* < 0.0001) (Fig. 1f). The proportion of HVA to DOPAC was decreased from 3.78 ng/mg wet tissue in NC tissue, to 1.15 in PD tissue (Fig. 1g).

Reduced levels of HVA is indicative of reduced catechol-*O*-methyltransferase (COMT)-mediated metabolism of dopamine [33]. COMT protein levels, measured via ELISA showed a significant increase in the PD olfactory bulb (*P* = 0.01). COMT activation occurs in a stepwise manner; inactive COMT must sequentially bind 1: *S*-adenosylmethionine (SAMe); 2: magnesium ion; 3: dopamine/DOPAC. In PD tissue, there is a reduction in *S*-adenosylmethionine (SAMe; *P* = 0.0003) and an increase in Mg^2+^ (*P* = 0.03) (Fig. 1h). A reduction in the substrate (SAMe) and the product (HVA) are consistent with decreased COMT activity in the olfactory bulb of PD tissue. There were no changes in monoamine oxidase (MAO) protein levels (Supplementary Fig. 2), or MAO activity detected in tissue lysate between PD and NC (Fig. 1i) and there was a significant increase in D_2_ receptor levels (*P* = 0.03) (Fig. 1j). Due to limitations with the amount of tissue available, the MAO activity assay was only able to detect changes in 8 NC and 6 PD samples, 6 samples fell below the limit of detection and were excluded from analysis. Full Western blot images are provided in Supplementary Fig. 3.

### Positive correlation between COMT and HVA is lost in Parkinson’s disease olfactory bulbs.

**Fig. 2:**
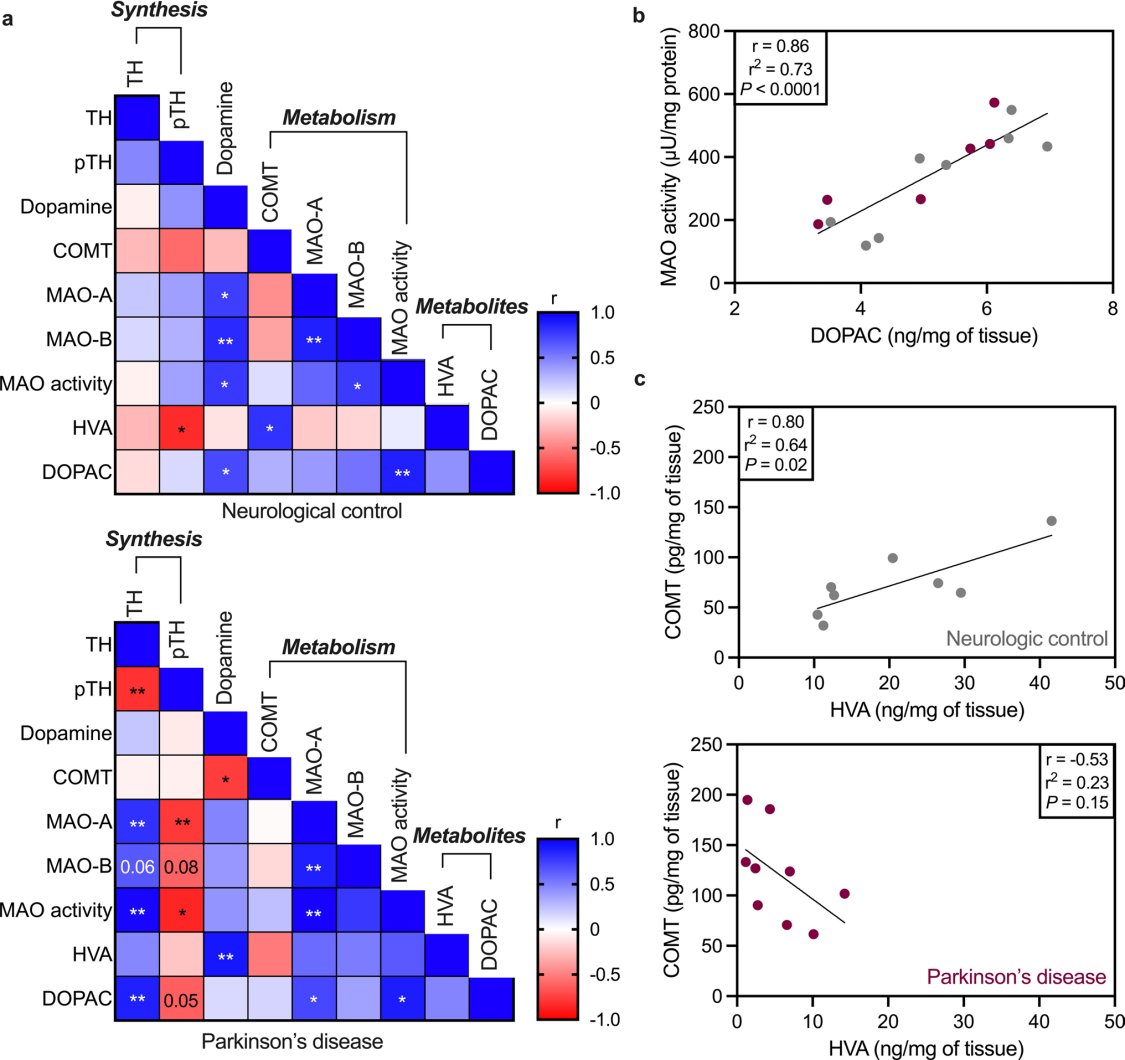
Correlation analysis between dopamine synthesis and metabolism in PD olfactory bulb. **a** Correlation matrix assessing the relation between key dopamine synthesis and metabolism enzymes, dopamine, and dopamine metabolites in neurological control (top) and Parkinson’s disease (bottom). The color scale depicts the strength of the Pearson correlation coefficient (r). A positive correlation (blue) indicates that the variables either increase or decrease in the same direction. A negative correlation (red) indicates that the variables increase or decrease in opposing directions. Asterisks depict the significance of the correlation: * *P* < 0.05, ** *P* < 0.01. **b** Positive correlation of monoamine oxidase (MAO) activity and 3,4-dihydroxyphenylacetic acid (DOPAC). **c** Correlation of catechol-*O*-methyltransferase (COMT) and homovanillic acid (HVA) in neurological controls (top) and Parkinson’s disease (bottom). Data passed normality testing (D’Agostino–Pearson test) and correlation was determined by Pearson correlations

Correlation analysis between key dopamine synthesis and metabolism enzymes with dopamine, DOPAC, and HVA levels revealed key changes in the PD olfactory bulbs (Fig. 2a). In both NC and PD tissue, as the level of MAO activity increases, the amount of DOPAC also increases (*P* = 0.005 and *P* = 0.02, respectively; NC and PD combined *P* < 0.0001) (Fig. 2b). In NC, as the level of COMT increases, the level of HVA increases (*P* = 0.02), however this positive correlation is lost in PD (Fig. 2c). Correlation matrices with superimposed individual r values are provided in Supplementary Fig. 4.

### Dopamine metabolism in altered in tau knockout mice

**Fig. 3:**
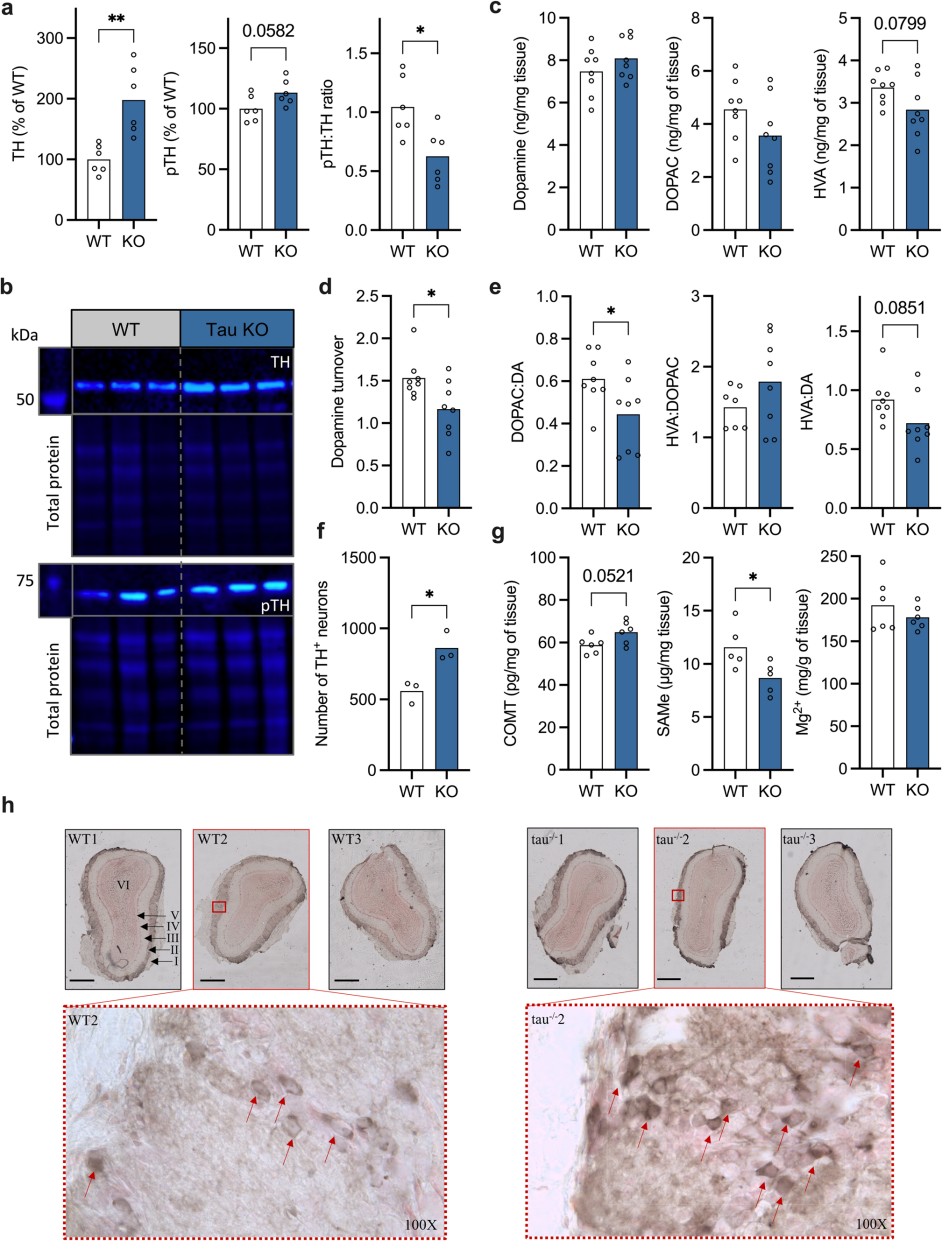
Dopamine metabolism is altered in the tau knockout olfactory bulb. **a** Quantification of TH and pTH immunoblot densitometry presented as % of neurological control, and ratio of pTH:TH. **b** Representative immunoblots of olfactory bulb lysates stained for tyrosine hydroxylase (TH), pTH (ser40) and total protein. **c** Dopamine, 3,4-dihydroxyphenylacetic acid (DOPAC), and homovanillic acid (HVA) concentration determined by HPLC-ED. **d** Dopamine turnover (DOPAC + HVA/dopamine). **e** Ratio of DOPAC:dopamine, HVA:DOPAC and HVA:dopamine in olfactory bulb tissue. **f** Quantification of TH^+^ neurons. **g** Catechol-*O*-methyltransferase (COMT) protein level, *S*-adenosylmethionine (SAMe) protein levels, and magnesium levels in tissue lysate. **h** Images of TH-stained sections from three WT and three tau KO olfactory bulbs, scale bar represents 500 µm; red arrows on 100X images indicate TH+ neurons. Analyses performed by Students’ T test; *P < 0.05, ** *P* < 0.01

To further investigate changes in dopamine metabolism in vivo, we interrogated dopamine metabolism and degradation in tau knockout mice (tau KO), which we have previously characterized as a model of the early olfactory deficit of PD [4]. The tau KO have increased α-syn and iron within the olfactory bulb [4, 6], consistent with human PD tissue in this study. Olfactory bulbs were isolated from 8-month-old mice, as we have previously reported that mice at this age have a substantial olfactory deficit but have not progressed to motor deficit [4]. Compared to WT controls, TH was increased (*P* = 0.003), pTH was also increased, although this did not reach significance (*P* = 0.06), and the ratio of pTH:TH was significantly decreased in tau KO animals (*P* = 0.02) (Fig. 3a and b). There was a significant increase in the number of TH positive (TH^+^) neurons in the tau KO olfactory bulb (*P* = 0.02), as determined by immunohistochemistry (Fig. 3c and d). Next, dopamine and metabolites DOPAC and HVA were analyzed with HPLC-ED. There was no difference in the concentration of dopamine or DOPAC between tau KO and WT controls, and there were reduced levels of HVA, however this did not reach significance (*P* = 0.08) (Fig. 3e). Like in human tissue, there was a shift from a positive correlation between COMT and HVA in WT mice, which was lost in tau KO, but this was non-significant (Supplementary Fig. 5). The ratios of dopamine and metabolites were assessed and there was a significant decrease in the ratio of DOPAC:dopamine (*P* = 0.045), and a reduction in HVA:dopamine, although this did not reach significance (*P* = 0.09) (Fig. 3f). There was a significant reduction in dopamine turnover in tau KO mice (*P* = 0.03) (Fig. 3g). Finally, COMT protein levels were increased (*P* = 0.05), SAMe levels were decreased (*P* = 0.04) and there was no change in Mg^2+^ in the olfactory bulb (Fig. 3h).

### Pharmacological modulation of dopamine availability in tau knockout mice

**Fig. 4:**
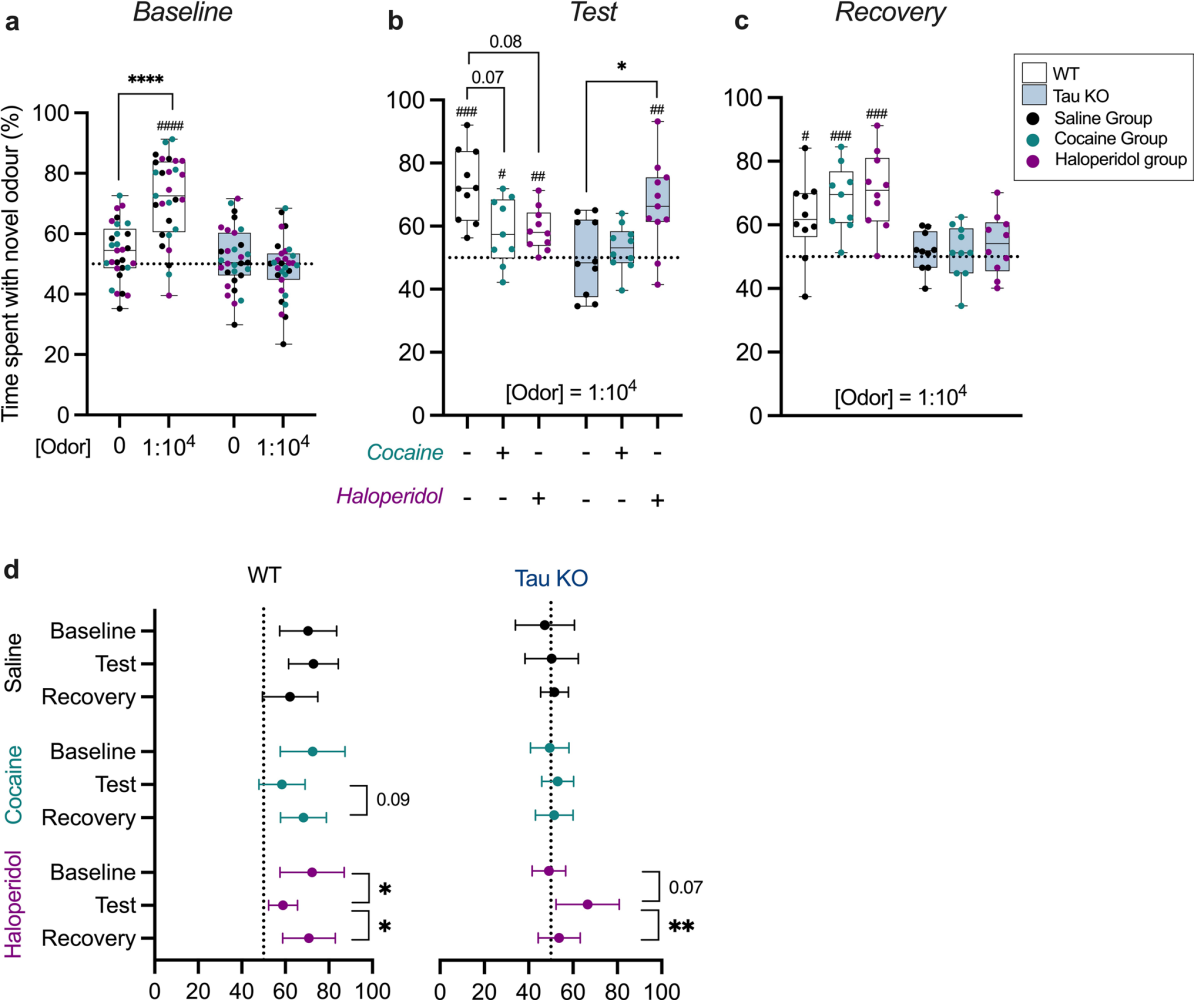
Dopamine receptor antagonism transiently restores olfactory deficit in tau knockout mice. **a** ‘Baseline’ odor detection test of WT (N = 29) and tau knockout (N = 31) mice performed at 8-months-old. **b** Seven days later, animals underwent a ‘test’ ODT 1 h after injection of saline (WT N = 10, tau KO N = 10), 20 mg/kg cocaine (WT N = 9, tau knockout N = 10), or 0.33 mg/kg haloperidol (WT N = 10, tau KO N = 11). **c** Following a 24-h drug washout period, animals underwent a ‘recovery’ ODT. **d** Inter-group comparisons of odor detection performance between test days. Analysis was performed by a three-way ANOVA with Sidak post hoc comparisons (denoted by asterisks), *P < 0.05, ****P < 0.0001. Secondary analysis was performed by a one-sample t-test with a hypothetical mean of 50% (denoted by octothorpes). ^#^*P* < 0.05, ^##^*P* < 0.01, ^###^*P* < 0.001, ^####^*P* < 0.0001

Tau KO mice have previously been reported to show an early olfactory deficit [4] and dopamine metabolism is altered in the olfactory bulb (Fig. 3). To test the potential of altered dopamine breakdown affecting olfaction, as would be the case with reduced COMT metabolism, tau KO and WT mice underwent a pharmacological dopamine challenge. To confirm an overt olfactory deficit in tau KO mice, animals underwent a baseline odor detection test (ODT). As expected, WT animals spent significantly more time with an odorous cannister compared to non-odorous cannister (Student’s t test; *P* < 0.0001) and compared to chance (one-sample t test; *P* > 0.0001); and tau KO mice were unable to detect novel odors (Fig. 4a).

Seven days later, mice received an i.p. injection of either cocaine (a competitive inhibitor of dopamine transporter—prolonging dopamine in the extracellular space [66]), or haloperidol (a dopamine receptor antagonist—preventing transmission of dopamine signal to the post-synaptic receptor) before undergoing an ODT. Cocaine and haloperidol reduced the odor detection score in WT mice, though this did not reach significance (*P* = 0.07 and *P* = 0.08, respectively, Fig. 4b). Despite this reduction, neither compound completely impaired olfaction in the WT mice, who spent significantly more time investigating novel odors compared to chance (one-sample t test; saline: *P* < 0.0001, cocaine: *P* = 0.04, haloperidol: *P* = 0.002). In the tau KO mice, saline and cocaine treatment did not affect olfaction, and mice remained anosmic. Conversely, haloperidol was able to rescue the olfactory phenotype, evidenced by haloperidol treated mice performing significantly better than saline control tau KO mice (Student’s t test, *P* = 0.02) and chance (one-sample t test, *P* = 0.003) (Fig. 4b). After a 24 h wash-out period, mice underwent a recovery ODT. All groups returned to baseline levels, with WT animals in saline, cocaine, and haloperidol groups performing significantly greater than chance (one-sample t test; *P* = 0.01, *P* = 0.0008, and *P* = 0.0004, respectively) (Fig. 4c).

Within groups, there were no significant changes in odor detection between baseline, test, and recovery in saline-treated WT or tau KO mice. In WT mice, there was a non-significant reduction in odor detection in cocaine-treated WT mice between test and recovery (*P* = 0.09), and haloperidol caused a significant reduction in odor detection (baseline vs. test, *P* = 0.03) that was recovered following wash-out (test vs. recovery, *P* = 0.04). In tau KO mice, there were no significant changes in olfaction in the cocaine group between tests, however haloperidol increased odor detection performance (baseline vs. test, *P* = 0.006), and animals returned to anosmic following wash-out (test vs. recovery, *P* = 0.07) (Fig. 4d).

## Discussion

Hyposmia is among the most common symptoms of Parkinson’s disease (PD), however there is limited understanding of the biochemical processes that underlie olfactory deficits [16, 69]. Studies on the post-mortem OB in PD remain limited, but existing research consistently shows that α-synuclein aggregates accumulate in the OB, especially in the anterior olfactory nucleus, and are associated with a decline in olfactory function. Recent investigations into molecular and cellular changes in the OB have revealed signs of increased oxidative stress, mitochondrial dysfunction, neurodegeneration, and neuroinflammation (including both microgliosis and astrogliosis) in PD [23]. A recent study also found that non-neuronal cells in the anterior olfactory nucleus, such as microglia, pericytes, and astrocytes, contain intracellular α-synuclein inclusions, suggesting these cells may contribute to the progression of PD [61]. Furthermore, iron and sodium levels are elevated in the OB, and free zinc is found to colocalize with α-synuclein aggregates, indicating that metal imbalances may play a role in the disease’s pathogenesis [24].

Contrary to the dopaminergic degeneration in the midbrain, dopaminergic neurons have been reported to increase in the olfactory bulb in PD [30, 45]. Dopamine is the inhibitory neurotransmitter between the glomeruli of the olfactory bulb and glomeruli are the first transmission relay point between the olfactory receptor neurons in the nasal cavity and the mitral cells of the olfactory bulb [41]. It was previously hypothesized that increased tyrosine hydroxylase (TH)-positive interneurons would result in increased dopamine levels, which inhibit olfactory transmission in the glomeruli [30]. Despite the increase in TH expression and the number of TH-positive periglomerular cells in our study, we found no difference in the concentration of dopamine between PD and neurological control (NC) samples, suggesting that TH is not synthesizing more dopamine despite increased expression of the enzyme.

In the synaptic cleft, dopamine interacts with post-synaptic receptors or presynaptic auto receptors [68, 72]. To stop signaling, extracellular dopamine needs to be cleared from the synaptic cleft rapidly, which occurs via reuptake [dopamine transporter (DAT)] and/or enzymatic degradation. In the first case, dopamine re-enters the presynaptic terminal via DAT where it is either recycled or intracellularly catabolized by monoamine oxidase (MAO) into 3,4-dihydroxyphenylacetic acid (DOPAC). Extracellular dopamine can be catabolized into homovanillic acid (HVA) in microglia, a reaction that is mediated by catechol-*O*-methyltransferase (COMT) [33]. There are regional preferences for which degradation pathway predominates throughout the brain. For example, in the striatum the ratio of HVA to DOPAC is 0.85, consistent with a DAT-MAO predominant degradation pathway. Conversely, in the olfactory bulb the HVA to DOPAC ratio is 2.18, reflective of a preference of COMT-mediated extracellular breakdown [21]. In this study, we report a ratio of HVA to DOPAC of 3.8 in NC, which is reduced to 1.2 in the PD tissue, driven by a significant reduction in the level of HVA, consistent with a reduction in COMT-mediated catabolism.

COMT is a Mg^2+^-dependent enzyme that transfers activated methyl groups from *S*-adenosylmethionine (SAMe) to catechol hydroxyl groups [42, 43]. We found a significant increase in the protein expression of COMT in the PD tissue, potentially compensatory. COMT activation occurs in a highly regulated manner, whereby inactive COMT binds SAMe, followed by a Mg^2+^ ion, then either dopamine or DOPAC. In the PD tissue there was a significant decrease in SAMe. The reduction in the methyl donor SAMe and HVA are reflective of diminished COMT activity. Decreased COMT-metabolism will result in increased extracellular dopamine, as is found in COMT KO mice who manifest higher spikes in dopamine, as they cannot rapidly clear synaptic dopamine [34]. Importantly, individuals with schizophrenia who carry the Val(158)Met *COMT* mutation have a reduction in odor identification accuracy, and TH is significantly increased in people with COMT-Val or COMT-Met mutations, considered to be a cellular response to suboptimal COMT activity—potentially explaining the increased levels of TH in the PD olfactory bulb [2, 35]. COMT inhibitors are frequently used as adjunctive therapy to extend the effects of l-DOPA in Parkinson’s disease. In this study, 4/10 PD donors were treated with entacapone. It is important to note that these drugs act peripherally, meaning they do not cross the blood–brain barrier and are not known to directly affect COMT activity in the brain [52].

As COMT, rather than DAT, is primarily responsible for the regulation of dopamine degradation in the olfactory bulb [10], we hypothesize that reduced COMT may result in increased residence time of dopamine on dopamine receptors. Extracellular dopamine will bind and activate post-synaptic D_2_R, and D_2_R levels are increased in the PD tissue in this study. D_2_R are the most abundant dopamine receptor subtype in the olfactory bulb [11], and they depress synaptic transmission between olfactory receptor neurons and mitral cells [29]. Activation of the D_2_R has been shown to both enhance and impair odor discrimination and pharmacological blockade or genetic ablation of the D_2_R impairs odor discrimination in rodents [22, 62, 63, 67]. Given our hypothesis that increased activation of the D_2_R, both pre- and post-synaptic, may underlie hyposmia in PD, we sought to test the hypothesis in a murine model of parkinsonism, the tau knockout (tau KO) mice. Tau KO mice present with an age-dependent loss of nigrostriatal neurons, α-syn and iron accumulation, and motor impairment [38–40], and we have previously demonstrated that these mice present with an olfactory deficit 5 months before the onset of motor impairment [4, 6]. To our knowledge, we are the first to report olfactory deficits in this model. The findings presented in this study further strengthen the face validity of this model as a relevant representation of PD-related hyposmia. In line with the findings in human tissue, we found a significant increase in the protein expression and number of TH positive neurons in tau KO olfactory bulbs. Additionally, there were alterations in the ratio of dopamine to its metabolites, a significant reduction in dopamine turnover, and reduced SAMe. These data suggest tau KO mice have altered dopamine processing in the olfactory system, potentially driven by the altered microtubule dynamics as a result of tau ablation, or in response to increased α-syn levels which is a known mediator of dopamine metabolism [70].

To test the hypothesis that extracellular dopamine levels contribute to dysfunctional olfaction, we intoxicated tau KO mice with cocaine, a DAT inhibitor that prolongs the duration of dopamine in the extracellular space and enhances dopamine release [25, 28, 32, 51, 66]. Despite the relatively low expression of DAT in the olfactory bulb, cocaine transiently reduced odor detection in WT mice. Increased dopamine availability resulting in hyposmia is supported by reports that cocaine can induce reversible hyposmia in humans [73]. Additionally, female mice have a surge of dopamine within the olfactory bulb directly after mating that seems to disrupt the perception of odors contained within male mouse urine [59]. Increased extracellular dopamine will activate post-synaptic D_2_ receptors (D_2_R) and inhibit neurotransmission and a previous study demonstrated that D_2_R agonists, such as quinpirole, reduced the odor detection performance in rats [18]. To test the role of D_2_R activation in PD-related hyposmia, WT and tau KO animals were treated with haloperidol, an antipsychotic with both high affinity and slow dissociation kinetics for the D_2_R [57]. This affinity for the D_2_R results in an accelerated turnover of dopamine [9, 46], and a low dose blockade of the D_2_R improves the ability of the adult rat to discriminate structurally and perceptually similar odors [71]. It is important to note that haloperidol acts on both the D_2_R and the D_2_ autoreceptor and it may have differential effects at different concentrations [15]. In this study, antagonizing the D_2_R’s of the WT mice reduced the odor detection, however, this may be due to a relatively high dose of haloperidol that caused an acute disruption of the system, as opposed to low dose long-term studies where the system was able to adapt. Importantly, haloperidol was able to transiently rescue the olfactory deficits in young tau KO mice, suggesting that the olfactory dysfunction in the tau KO animals is, at least in part, due to increased activation of the D_2_R, and olfaction can be restored by acute antagonism. This mechanism is further supported by Doty et al*.* who report that mice treated with quinpirole, a D_2_R agonist, develop a dose-dependent loss in olfaction, and pre- or post-treatment with the D_2_R antagonist spiperone mitigated this effect [18].

This study demonstrates altered COMT-mediated dopamine metabolism in the *post-mortem* PD olfactory bulbs. By integrating findings from a hyposmic animal model, we hypothesize that disruptions in dopamine dynamics may contribute to olfactory dysfunction in PD. Importantly, it must be considered that although acute antagonism was able to rescue the hyposmia in young tau KO mice, there was no effect in aged animals (15 months old, data not shown). The age-dependence of intervention suggests that there may be metabolic perturbations that occur early in the disease process that can be rescued pharmacologically, but as the disease progresses there are different biological and/or anatomical changes that underlie dysfunction. The olfactory bulb plays a crucial role as the first stage in olfactory processing, where it detects and begins encoding odorant signals. However olfactory perception, emotional response, and memory relies on the integration of the olfactory bulb’s input with other brain regions, such as the piriform cortex, amygdala, hippocampus, and orbitofrontal cortex. Together, these areas work to transform the raw sensory data from the olfactory bulb into complex cognitive and emotional experiences associated with odor. Early neuropathological changes in the olfactory bulb and anterior olfactory nucleus (AON) are observed in PD, followed by alterations in the olfactory cortex and limbic structures as the disease progresses. While our data demonstrates changes at the olfactory bulb level, there are many other olfactory regions that are yet to be explored thoroughly in the context of PD.

While some studies have suggested alterations in dopaminergic signalling in the olfactory bulb, this remains a relatively understudied area, and further research is needed to clarify the role of TH and other dopaminergic markers in early PD pathology. Additionally, while our findings provide valuable insights, they would benefit from validation in larger neuropathological datasets and other PD animal models. Larger studies could help confirm these results, investigate regional differences, and further elucidate the mechanisms underlying olfactory dysfunction in PD. Another important consideration is the potential involvement of other neurotransmitter systems in olfactory processing, such as the cholinergic system, which has been implicated in both olfactory function and PD pathology. Future studies should investigate these systems to provide a more comprehensive understanding of the neurochemical changes occurring in the olfactory bulb in PD. Finally, the relatively small sample size of our study limits the ability to detect sex and age-related differences in olfactory bulb pathology. Larger, well-powered studies are needed to explore how these variables may influence disease progression, as well as to investigate sex and age differences in olfactory dysfunction and the broader neurodegenerative processes in PD. Addressing these limitations will be crucial for advancing our understanding of the early olfactory involvement in PD and its potential as a diagnostic and therapeutic target.

## Supplementary Information

Below is the link to the electronic supplementary material.

Supplementary file1 (DOCX 1221 KB)
