# Supplementary material for: Evidence of COMT dysfunction in the olfactory bulb in Parkinson’s disease
Source: Acta Neuropathol. 2025 Mar 1;149(1):21. doi: 10.1007/s00401-025-02861-y (PMC11872990; doi:10.1007/s00401-025-02861-y)
Supplement: Supplementary file 1 — Supplementary file1 (DOCX 1221 KB) [file 401_2025_2861_MOESM1_ESM.docx]

**SUPPLEMENTARY**

**Supplementary Table 1. Antibodies**

| **Application** | **Target** | **Dilution** | **Company** | **Catalogue #** |
| --- | --- | --- | --- | --- |
| Western blot | TH | 1:5000 | CST | 2792S |
|  | pTH | 1:1000 | Abcam | ab51206 |
|  | α-synuclein | 1:5000 | BD Biosciences | 610786 |
|  | DJ1 | 1:1000 | Abcam | ab76008 |
|  | MAO-A | 1:1000 | Abcam | ab126751 |
|  | MAO-B | 1:1000 | Abcam | ab175136 |
| IHC | TH | 1:1000 | Millipore | AB152 |
|  | α-synuclein | 1:5000 | Abcam | ab138501 |

**Supplementary Table 2. Donor characteristics**

|  | **Age** | **Sex** | **PMD (hours)** | **Cause of Death** | **Tissue pH** |
| --- | --- | --- | --- | --- | --- |
| PD | 84 | M | 5 | Cholangiocarcinoma | 6.4 |
|  | 82 | M | 19 | Cardiorespiratory failure | 6.4 |
|  | 81 | F | 29 | Cardiorespiratory failure | 6.4 |
|  | 71 | M | 25 | Hypertensive heart disease | 6.5 |
|  | 82 | F | 9 | Pneumonia | 6.1 |
|  | 92 | M | 46 | Cardiorespiratory failure | 6.1 |
|  | 93 | F | 9 | Cardiorespiratory failure | 6.1 |
|  | 78 | M | 26 | Cardiorespiratory failure | 5.6 |
|  | 80 | F | 21 | Cardiorespiratory failure | 6.0 |
|  | 88 | F | 21 | Cardiorespiratory failure | 6.3 |
| NC | 79 | M | 8 | Pulmonary embolism | 7.5 |
|  | 89 | F | 23 | Metastatic adenocarcinoma | 6.0 |
|  | 88 | F | 31 | Congestive cardiac failure | 6.2 |
|  | 84 | M | 22 | Cardiorespiratory failure | 5.8 |
|  | 84 | M | 36 | Severe pulmonary hypertension | 6.4 |
|  | 80 | F | 29 | Cardiorespiratory failure | 6.3 |
|  | 73 | M | 39 | Cardiorespiratory failure | 6.3 |
|  | 88 | M | 9 | Cardiorespiratory failure | 6.4 |
|  | 85 | F | 10 | Cardiorespiratory failure | 6.6 |
|  | 86 | F | 4 | Cardiorespiratory failure | 6.4 |

F, female; NC, neurological control; M, male; PD, Parkinson’s disease; PMD, post-mortem delay.

**
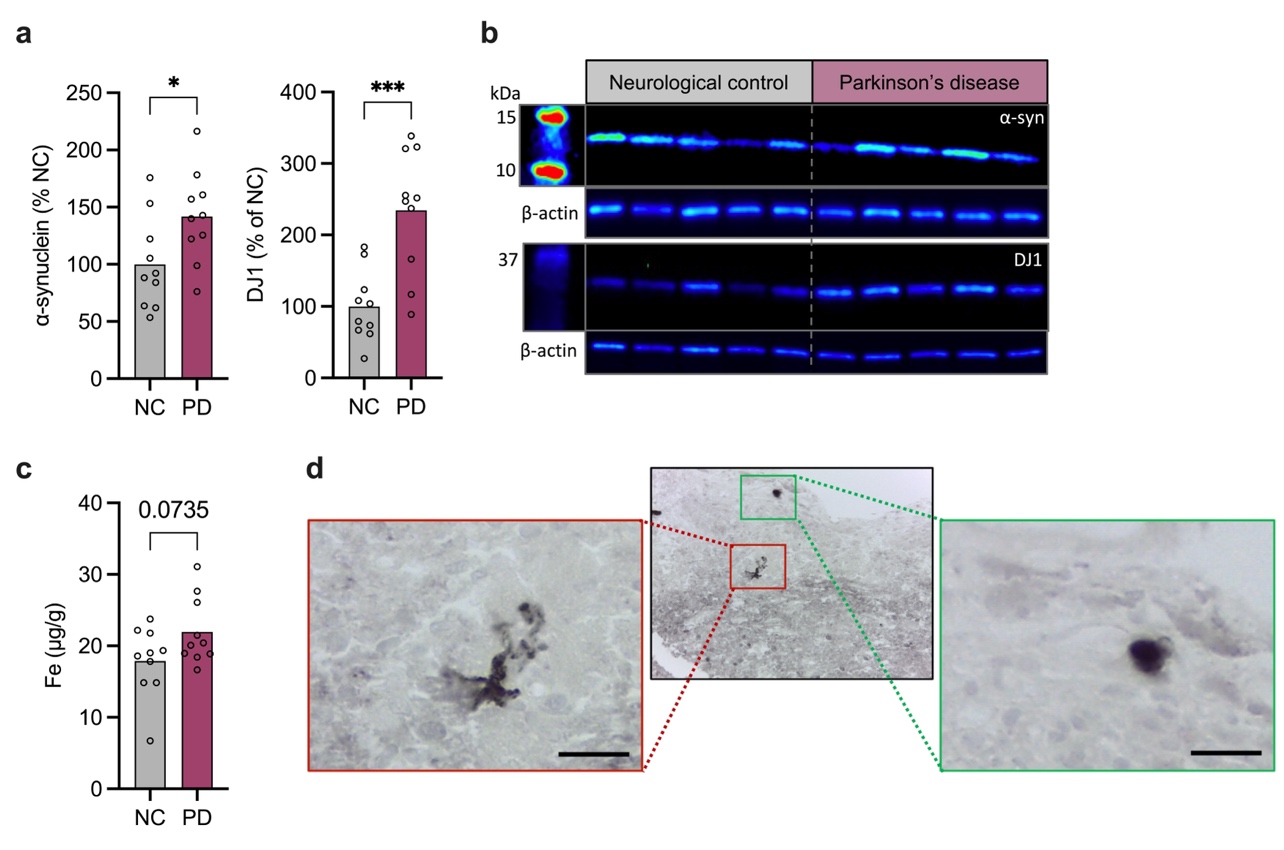
**

**S Fig 1** Parkinson’s disease pathology in the olfactory bulb **a** Quantification of α-synuclein and DJ1 immunoblot densitometry presented as percentage of neurological control, a-syn increased by 41.8±17.9%, DJ1 increased by 134.7±31.4%. **b** Representative immunoblots of olfactory bulb lysates. **c** Iron (Fe) ICP-MS analysis, Fe increased by 4.1±2.1 μg/g. **d** Representative images of the α-synuclein pathology found within the PD olfactory bulb; scale bar represents 40 μm. NC (n=10) and PD (n=10). Analyses performed by Students’ T Test; **P*<0.05, ****P*<0.001

**
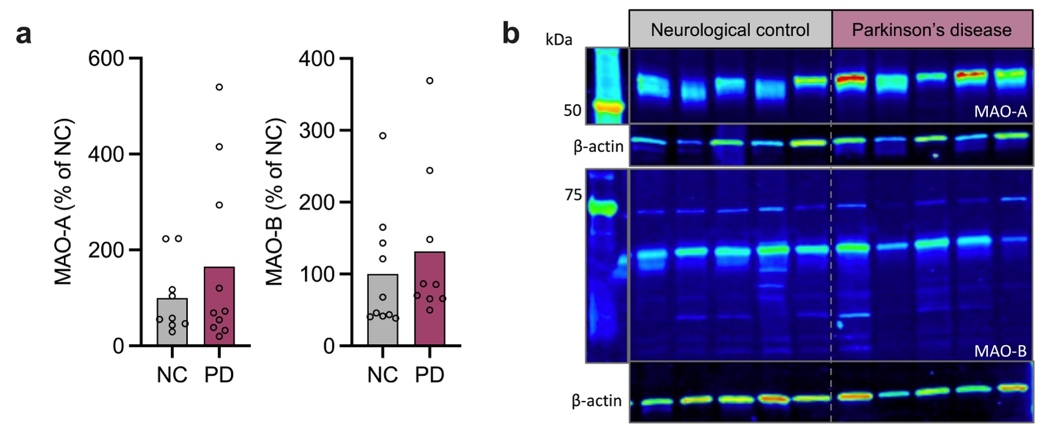
**

**S Fig 2** Monoamine oxidase is not altered in Parkinson’s disease. **a** Quantification of MAO-A and MAO-B immunoblot densitometry presented as % of neurological control. **b** Representative immunoblots of olfactory bulb lysates stained for MAO-A, MAO-B and β-actin.

**
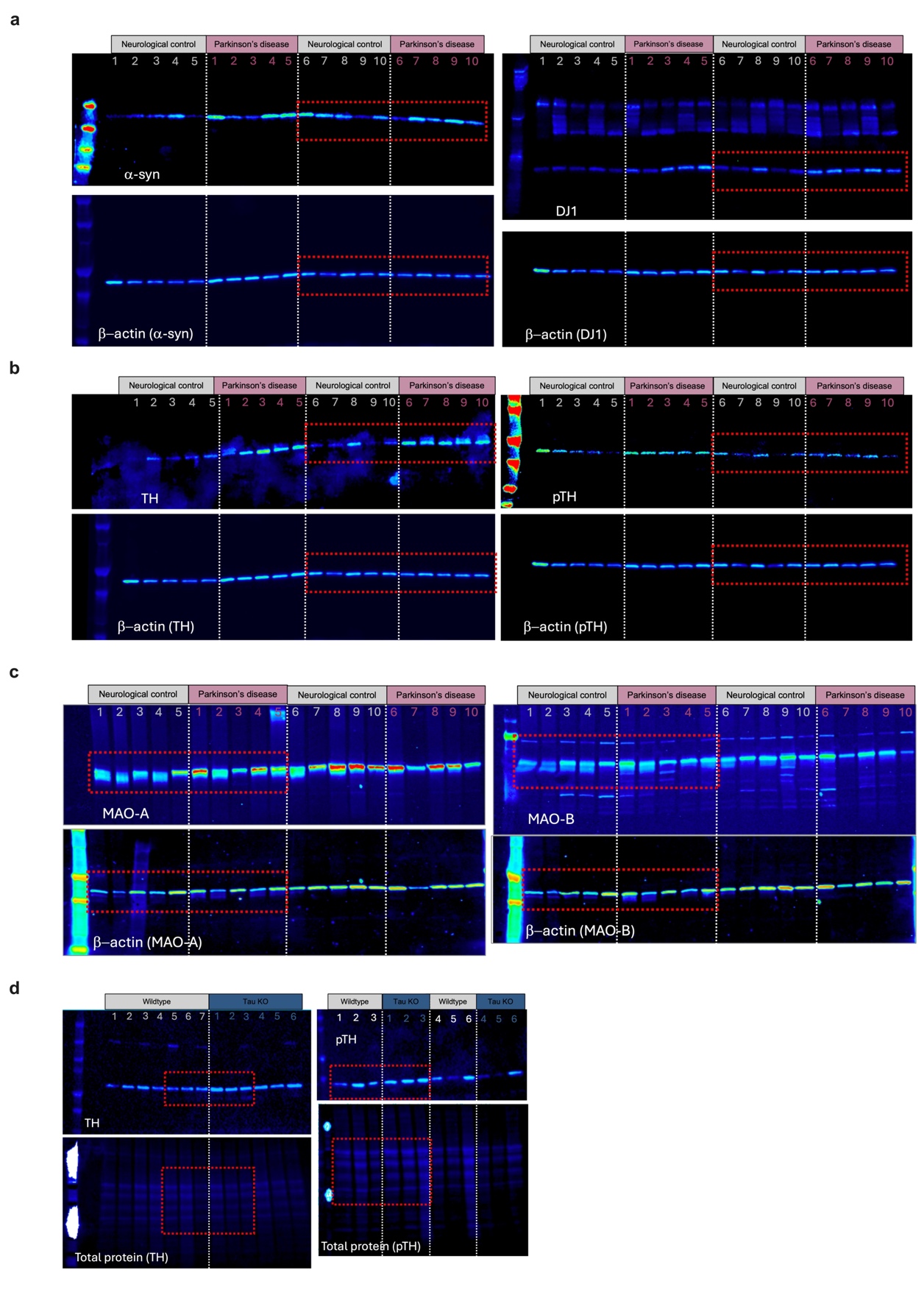
**

**S Fig 3** Full Western blot images **a** Alpha-synuclein and DJ1 in neurological control and Parkinson’s disease olfactory bulb lysate; Supplementary Figure 1. **b** Tyrosine hydroxylase and phosphor-Ser40 tyrosine hydroxylase in neurological control and Parkinson’s disease olfactory bulb lysate; Figure 1. **c** Monoamine-A and monoamine-B in neurological control and Parkinson’s disease olfactory bulb lysate; Supplementary Figure 2. **d** Tyrosine hydroxylase and phosphor-Ser40 tyrosine hydroxylase in wildtype and tau knockout mouse olfactory bulb lysate; Figure 3. Red box indicates cropped samples presented in manuscript figures.

**
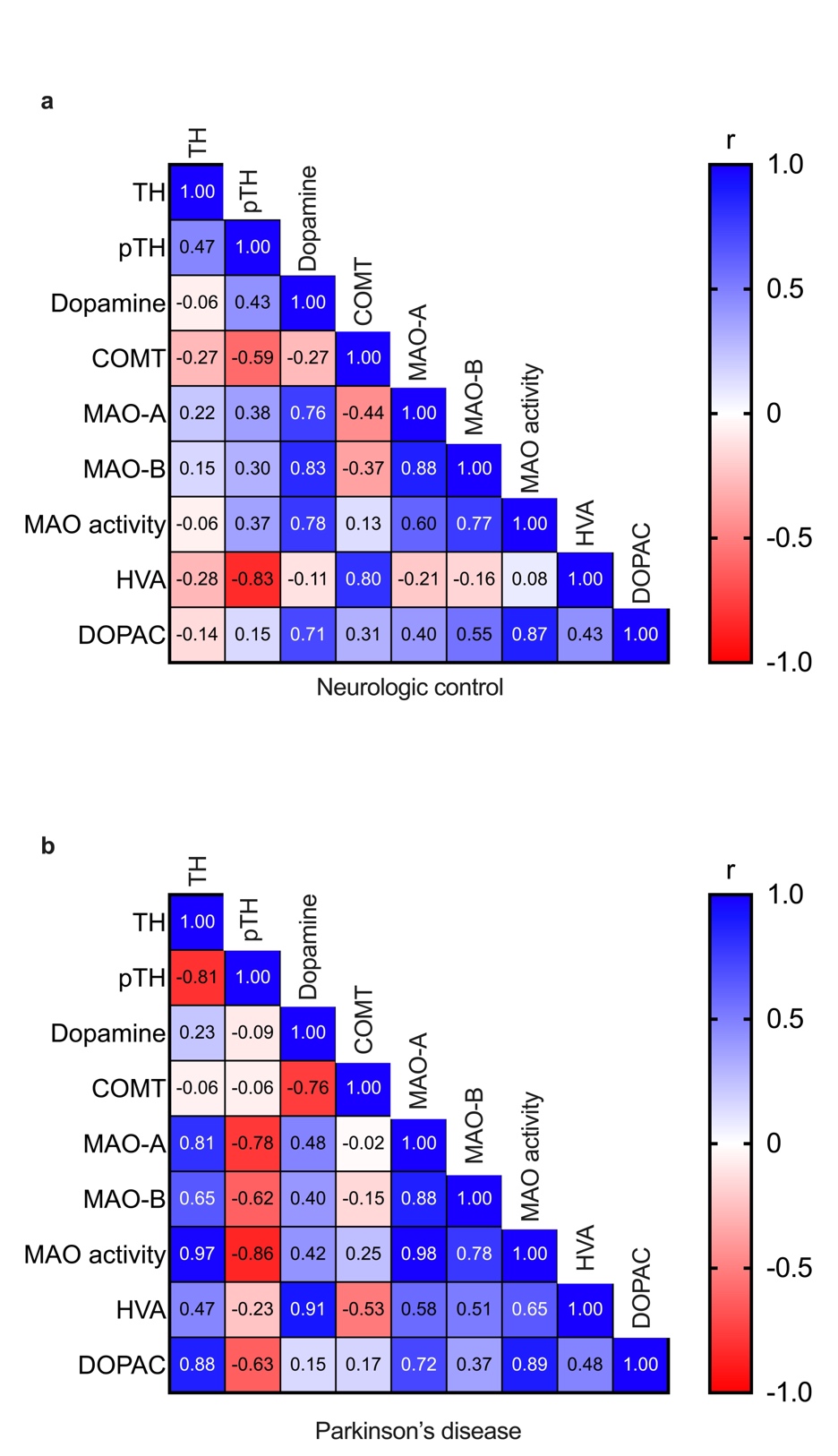
**

**S Fig 4** Correlation analysis between dopamine synthesis and metabolism in the olfactory bulb. **a** Correlation matrix assessing the relation between key dopamine synthesis and metabolism enzymes, dopamine, and dopamine metabolites in neurological control and **b** Parkinson’s disease. The color scale depicts the strength of the Pearson correlation coefficient (r), which is superimposed as text on the matrix.

**
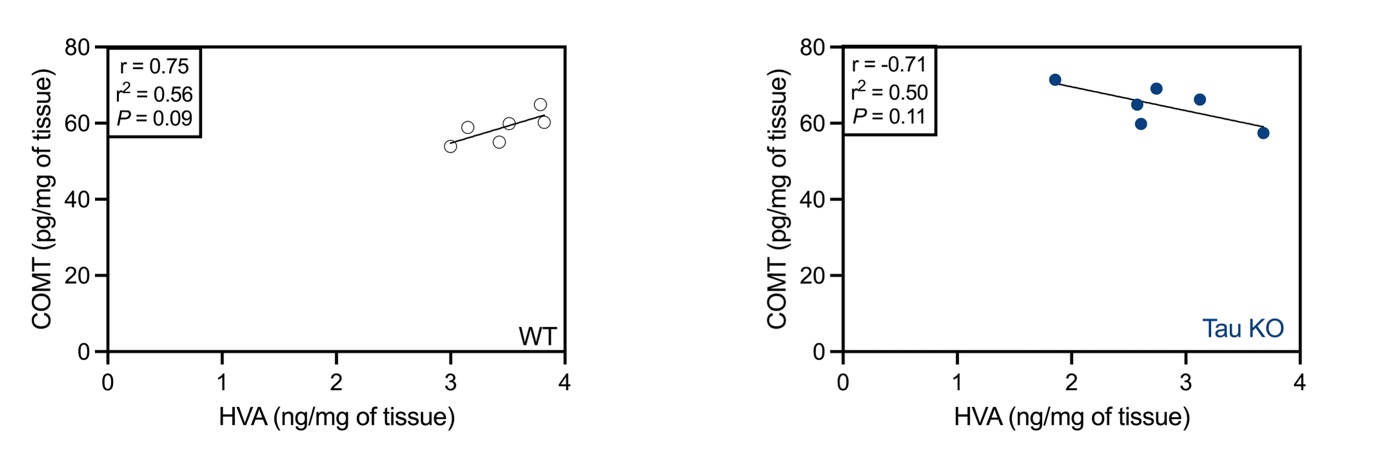
**

**S Fig 5** Correlation of catechol-*O*-methyltransferase (COMT) and homovanillic acid (HVA) in wildtype (left) and tau knockout (right) olfactory bulb lysate.
